# PYR-41 and Thalidomide Impair Dendritic Cell Cross-Presentation by Inhibiting Myddosome Formation and Attenuating the Endosomal Recruitments of p97 and Sec61 via NF-*κ*B Inactivation

**DOI:** 10.1155/2018/5070573

**Published:** 2018-07-05

**Authors:** Xiang You, Dan Dan Xu, Di Zhang, Jie Chen, Feng Guang Gao

**Affiliations:** ^1^Department of Immunology, Basic Medicine Science, Medical College, Xiamen University, Xiamen, Fujian 361102, China; ^2^State Key Laboratory of Oncogenes and Related Genes, Shanghai Jiao Tong University, Shanghai 200032, China

## Abstract

PYR-41 and thalidomide have therapeutic effects on inflammation-associated diseases with side effects such as tumorigenesis. Cross-presentation allows dendritic cells (DC) to present endogenous antigen and induce protective immunity against microbe infection and tumors. But, up to now, the effects of PYR-41 and thalidomide on cross-presentation are still uncertain. In this study, we investigated the effect and mechanism of PYR-41 and thalidomide on DC cross-presentation by observing Myddosome formation, endosomal recruitment of p97 and Sec61, NF-*κ*B activation, and cross-priming ability. We demonstrated that the inhibition of endosomal recruitment of p97 and Sec61, together with attenuated NF-*κ*B activation and Myddosome formation, contributes to PYR-41- and thalidomide-impaired cross-presentation and thereby reverses cross-activation of T cells. These observations suggest that NF-*κ*B signaling and p97 and Sec61 molecules are candidates for dealing with the side effects of PYR-41 and thalidomide.

## 1. Introduction

Ubiquitination-proteasome is responsible for the degradation of I*κ*B and regulation of NF-*κ*B activity [1]. PYR-41, an inhibitor of ubiquitin-activating enzyme E1, was reported to prevent the reduction of I*κ*B protein from degrading, suggesting the potential issues for E1 inhibitors as therapeutics in cancer [2]. Thalidomide, an inhibitor of E3 ubiquitin ligases, increased the degradation of TNF*α* mRNA and reduced the production of TNF*α*, making thalidomide a good candidate for the treatment of multiple myeloma [3]. Therefore, targeting ubiquitination by PYR-41 or thalidomide to inhibit NF-*κ*B activation may represent a potential strategy for the treatment of Crohn's disease and rheumatoid arthritis. Despite that PYR-41 and thalidomide have therapeutic properties, they still reveal severe side effects, such as tumorigenesis and angiogenesis [4, 5]. In addition to classical MHC I-restricted endogenous antigen presentation, cross-presentation allows dendritic cells (DC) presenting extracellular antigen and inducing protective immunity against intracellular microbe infection and tumors [6–8]. The side effects of PYR-41 and thalidomide indicate that these drugs might impair DC cross-presentation.

Cross-presentation occurs in the vacuolar and endosome-to-cytosol pathways, which degrades antigens within endosomes by lysosomal proteases or in the cytosol by cytosolic proteinase, respectively [7–10]. In the endosome-to-cytosol pathway, the internalized antigens need to be exported from the endosomes into the cytosol [9, 10]. During this process, p97, an AAA-ATPase that provides the driving force for the transport of misfolded proteins [11], translocated toward endosomes [8, 12, 13]. Sec61, another ER component protein that imports proteins into the ER, was also reported to relocate from the ER toward phagosomes during cross-presentation [14, 15]. Nevertheless, the inhibition of Sec61 impairing antigen cross-presentation independently of endosome-to-cytosol export was also documented [16]. But, up to now, little is known about the effects of PYR-41 and thalidomide on the endosomal translocation of p97 and Sec61.

Toll-like receptor 4 (TLR4) signaling, which mediates the recruitment of myeloid differentiation factor 88 (MyD88) and results in I *κ*B kinase degradation, was found to play a vital role in cross-presentation [15, 17–19]. For example, TLR4-MyD88 signaling mediated the endosomal relocation of transporter associated with antigen processing (TAP) and Sec61 to allow the entry of antigenic peptides [15, 17, 18]. Upon activation, MyD88 and IRAK4 were recruited to the endosomes to form myddosome (TLR4-MyD88-IRAK4) [20, 21]. Despite that the inhibition of NF-*κ*B by PYR-41 and thalidomide was already documented [2–4], the effects of PYR-41 and thalidomide on myddosome formation are still to be elucidated.

In this study, we investigated the mechanism of PYR-41 and thalidomide on cross-presentation by observing myddosome formation, endosomal recruitment of p97 and Sec61, inhibition of NF-*κ*B, and cross-priming ability. We demonstrated that PYR-41 and thalidomide attenuate LPS-induced NF-*κ*B activation. The inhibition of myddosome formation and endosomal recruitment of p97 and Sec61 lead to PYR-41- and thalidomide-induced impairment of cross-presentation and thereby reverse cross-priming. These observations suggest that NF-*κ*B signaling and p97 and Sec61 molecules are potential candidates for dealing with the side effects of PYR-41 and thalidomide.

## 2. Material and Methods

### 2.1. Mice

Pathogen-free C57BL/6 mice (female, 4–6 weeks old) were bought from the Shanghai Laboratory Animal Center of the Chinese Academy of Sciences and kept at the Animal Center of Xiamen University. The protocol was approved by the Ethics Committee of Animal Experiments of Xiamen University. All surgeries were performed under sodium pentobarbital anesthesia, and all efforts were made to minimize suffering.

### 2.2. Reagents and Antibodies

Reagents were purchased from the following companies: albumin from chicken egg white (OVA), LPS from *Escherichia coli*, and DAPI were obtained from Sigma-Aldrich (St. Louis, MO, USA). Recombinant mouse GM-CSF and IL-4 were obtained from PeproTech (Rocky Hill, NJ, USA). RPMI 1640 medium and fetal bovine serum were purchased from Hyclone (Logan, UT, USA). OVA peptide SIINFEKL of amino acids 257~264 were synthesized by Auspep (Tullamarine, VIC, Australia). PYR-41 and thalidomide were bought from Selleck Chemicals (Houston, TX, USA). PE-conjugated anti-mouse H2Kb bound to SIINFEKL (25-D1.16) was obtained from BioLegend (San Diego, CA, USA). Antibodies to TLR4 (D8L5W), MyD88 (D80F5), Rab7 (D95F2), phospho-NF-*κ*B p65 (93H1), I*κ*B*α* (L35A5), and GAPDH were bought from Cell Signaling Technology (Beverly, MA, USA). Antibodies to EEA1 (E-8), Rab5 (D-11), Sec61*α* (G-20), Sec61*β* (E-6), and calnexin (AF-18) were bought from Santa Cruz Biotechnology (Dallas, TX, USA); antibody to p97 (ABIN681178) was obtained from ABNOVA (Taipei, Taiwan); antibodies to Rabbit IgG (Chromeo 546), Rabbit IgG (Chromeo 488), mouse IgG (Cy3), and mouse IgG (Alexa Fluor) were from Abcam (Cambridge, UK). BrdU cell proliferation assay kit was obtained from Roche (Roche Diagnostics GmbH, Germany); IL-12 p70 ELISA kit (BMS6004) was obtained from eBioscience (San Diego, CA, USA); IFN-*γ* ELISPOT kit was obtained from U-CyTech biosciences (Utrecht, Netherlands). The PrimeScript RT-PCR kit and SYBR Premix Ex Taq™ kit were purchased from Takara Bio (Dalian, Liaoning, China).

### 2.3. Generation of Murine Bone Marrow-Derived DC

Bone marrow-derived DC were generated by culturing progenitors in RPMI 1640 medium supplemented with 30 ng/ml GM-CSF and 1 ng/ml IL-4 [22]. Nonadherent cells were gently washed out with PBS on day 4 of culture; the remaining loosely adherent clusters were used as semimatured DC. Cells were synchronized by serum starvation (in RPMI 1640 with 0.5% FBS) for 3 h prior to further treatment.

### 2.4. DC Treatment

To determine the effects of PYR-41 and thalidomide on cross-presentation and the endosomal recruitment of p97 and Sec61, the DC was treated with thalidomide (30 *μ*M) or PYR-41 (5 *μ*M) prior to ovalbumin pulse (50 *μ*g/ml). To investigate the effects of PYR-41 and thalidomide on LPS-induced NF-*κ*B activation and myddosome formation, the DC conferred PYR-41 or thalidomide pretreatment prior to LPS stimulation (10 ng/ml).

### 2.5. Flow Cytometric Measurements

The effect of PYR-41 or thalidomide on cross-presentation was determined via flow cytometry [23]. Flow cytometry was performed with FACSCalibur and data were analyzed with CellQuest software.

### 2.6. Confocal Immunofluorescence Microscope

Immunofluorescence observation was performed according to previous description [24]. Briefly, DC conferred PYR-41 or thalidomide treatment. Then, the cells were fixed in 2% PFA and permeabilized with 0.2% saponin. The cells were blocked, washed, and stained with primary antibodies overnight at 4°C. Fluorescence conjugated secondary antibodies were incubated for 1 h at 37°C. DAPI counterstaining was performed to visualize the nuclei. Images were acquired on Olympus FluoView FV1000 confocal microscope with oil immersion objective at the wavelength of 488 nm.

### 2.7. Mixed Lymphocyte Reaction Assays

Antigen-specific T cell proliferation assays were performed as previously described [25]. Briefly, DC conferred PYR-41 or thalidomide treatment prior to ovalbumin pulse and referred to stimulator cells. Responder cells were prepared by the depletion of red blood cells from splenocytes of the same H-2 background C57BL/6 mice. Then, the stimulator cells were mixed with responders at a ratio of 1 : 10 in 200 *μ*l volume. After 5 d coincubation, DC-dependent T cell proliferation was determined via BrdU cell proliferation assays.

### 2.8. IL-12 Enzyme-Linked Immunosorbent Assays

To access Th1 cell differentiation in the mixed lymphocyte reaction, the supernatants were collected and IL-12 concentration was determined by enzyme-linked immunosorbent assay (ELISA) according to the manufacturer's guideline.

### 2.9. Ag-Specific IFN-*γ* ELISPOT Assays

To access the effects of PYR-41 and thalidomide on cross-priming, antigen-specific IFN-*γ* ELISPOT assays were performed according to previous description [26]. Briefly, DC firstly conferred PYR-41 or thalidomide treatment prior to ovalbumin pulse. Then, 1 × 10^4^ cells were intraperitoneally transferred to C57BL/6 mouse in 100 *μ*l volume. 5 d after that, splenocytes of receipts were isolated and then placed into plates precoated with IFN-*γ* antibody (5× 10^5^ per well ). After 16~20 h SIINFEKL peptide stimulation at the final concentration of 2 *μ*g/ml, the spots were developed and the data were presented as spot-forming units per million cells.

### 2.10. Western Blots

The whole protein of PYR-41- or thalidomide-treated DC was obtained and NF-*κ*B activation was determined according to previous description [27]. Briefly, cell lysates were subjected to 10% SDS-PAGE and transferred onto a PVDF membrane (millipore). Membranes were blocked with 5% evaporated milk in Tris base SDS 0.05% Tween and were incubated with primary antibodies and peroxidase-conjugated secondary antibodies. Bound antibodies were revealed using the ECL Western blot reagents (Advansta, CA) according to the manufacturer's directions.

### 2.11. Quantitative Real-Time PCR

The expressions of granzyme B in the spleen and lymph node were investigated by RT-qPCR analysis. Briefly, total RNA was isolated from cells. Reverse transcription was performed using PrimeScript Reverse Transcriptase kit (Takara) and cDNA was used for subsequent real-time PCR reactions. Quantitative real-time PCR was conducted on an ABI Prism 7500 instrument using the Maxima SYBR green qPCR Master Mix (Takara). The cycling parameters were 95°C for 30 s, followed by 40 cycles of 95°C for 5 s and 60°C for 34 s; each assay was performed in triplicate, and the relative expression levels (defined as fold changes) of the target genes were normalized. The following primers were used: *β*-actin (sc-108070-PR) and granzyme B (sc-35508-PR) (Santa Cruz). However, primer sequences are not provided by Santa Cruz, as stated in their datasheets: “semi-quantitative RT-PCR may be performed to monitor gene expression knockdown using RT-PCR primer: *β*-actin (m)-PR: sc-108070-PR (600 bp) and granzyme B (m)-PR: sc-35508-PR (536 bp)”.

### 2.12. Statistical Analysis

All data were expressed as average of experimental data points, and standard error means were determined using the calculated standard deviation. Statistical significance was assessed by one-way ANOVA with the Newman–Keuls post test, with a value of *p* < 0.05 considered statistically significant.

## 3. Results

### 3.1. Treatment with PYR-41 or Thalidomide Impairs DC-Dependent Cross-Priming

**Figure 1:**
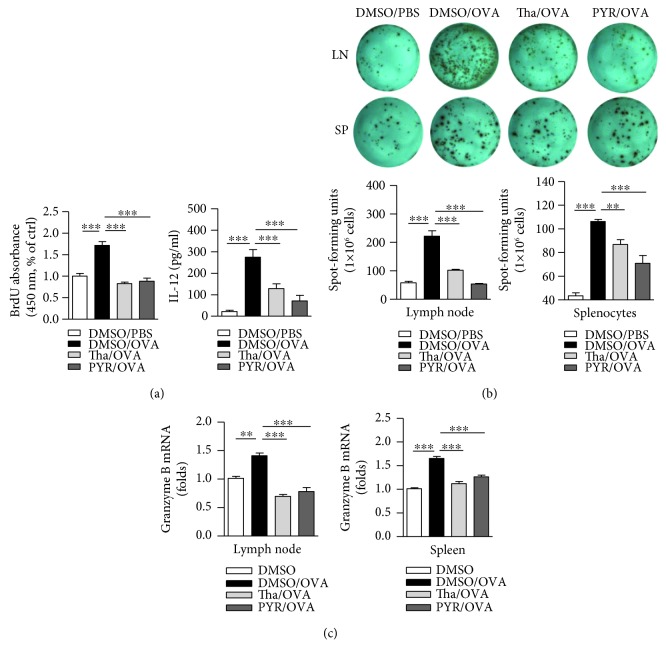
Treatment with PYR-41 or thalidomide impairs DC cross-priming. Murine bone marrow-derived DC (cultured for 4 d) conferred thalidomide (30 *μ*M), PYR-41 (5 *μ*M), or DMSO treatment prior to ovalbumin (50 *μ*g/ml) or PBS pulse. (a) Mixed lymphocyte reaction was performed by incubating these cells with the same H-2 background splenocytes at the ratio of 1 : 10. The ability of T cell proliferation was accessed by BrdU cell proliferation assay. IL-12 concentration in supernatants of mixed lymphocyte reaction was determined by ELISA. (b) C57BL/6 mouse was intraperitoneally transferred with these 1 × 10^4^ cells and Ag-specific IFN-*γ* ELISPOT assays, and (c) granzyme B expression determination was performed 5 d after adoptive transfer. The ELISPOT data were presented as spot-forming units per million cells. For qPCR, *β*-actin was used as an internal control. Data were presented as the mean ± SEM, ^∗∗^ *p* < 0.01, ^∗∗∗^ *p* < 0.001, and one-way ANOVA with Newman–Keuls post test. Tha: thalidomide; PYR: PYR-41.

Antigen-presenting cells, especially DC, uptake and process antigens and present them to T cells to prime series of immune response [6]. We firstly incubated DC with PYR-41 or thalidomide and found that PYR-41 and thalidomide impaired cell viability in a dose-dependent manner (Supplementary Materials description). Mixed lymphocyte reaction was performed by incubating PYR-41- or thalidomide-treated DC with T lymphocytes, and the results showed that the treatment with PYR-41 or thalidomide obviously impaired DC-dependent T cell proliferation (Figure 1(a)). ELISA assays revealed that the treatment with PYR-41 or thalidomide efficiently inhibited IL-12 secretion in the supernatant of mixed lymphocyte reaction (Figure 1(a)).

We next accessed the effects of PYR-41 and thalidomide on DC-dependent cross-priming by IFN-*γ* ELISPOT assay (Figure 1(b)) and granzyme B expression with qPCR (Figure 1(c)). Compared to normal splenocyte control, the transfer of ovalbumin-pulsed DC obviously increased the numbers of antigen-specific IFN-*γ* spot (Figure 1(b)) and granzyme B expression (Figure 1(c)) in both splenocytes and lymph nodes. Nevertheless, the treatment with PYR-41 or thalidomide efficiently abolished the effect of ovalbumin on DC-dependent CTL priming (Figures 1(b) and 1(c)).

**Figure 2:**
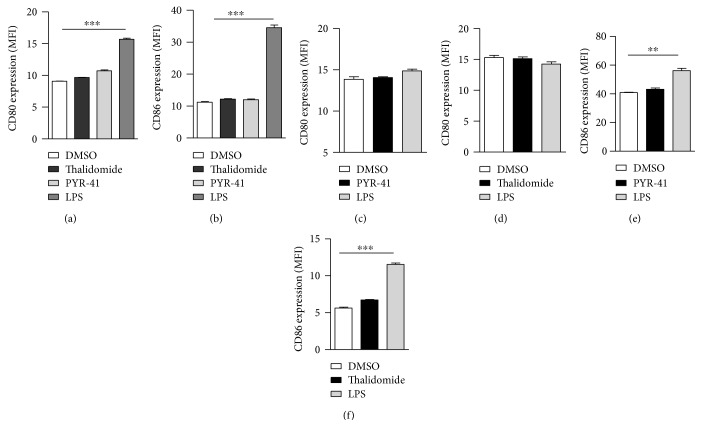
PYR-41 and thalidomide do not increase CD80/CD86 expression in long-term- and short-term-treated conditions. Murine bone marrow-derived DC (cultured for 4 d) conferred PYR-41 (5 *μ*M), thalidomide (30 *μ*M), LPS (10 ng/ml), or DMSO stimulation for 12 (a, b) or 2 (c–f) hrs. The expressions of CD80 and CD86 were determined by flow cytometry. Data were presented as the mean ± SEM, ^∗∗^ *p* < 0.01, ^∗∗∗^ *p* < 0.001, and one-way ANOVA with Newman–Keuls post test. One representative from 3 independent experiments was shown.

**Figure 3:**
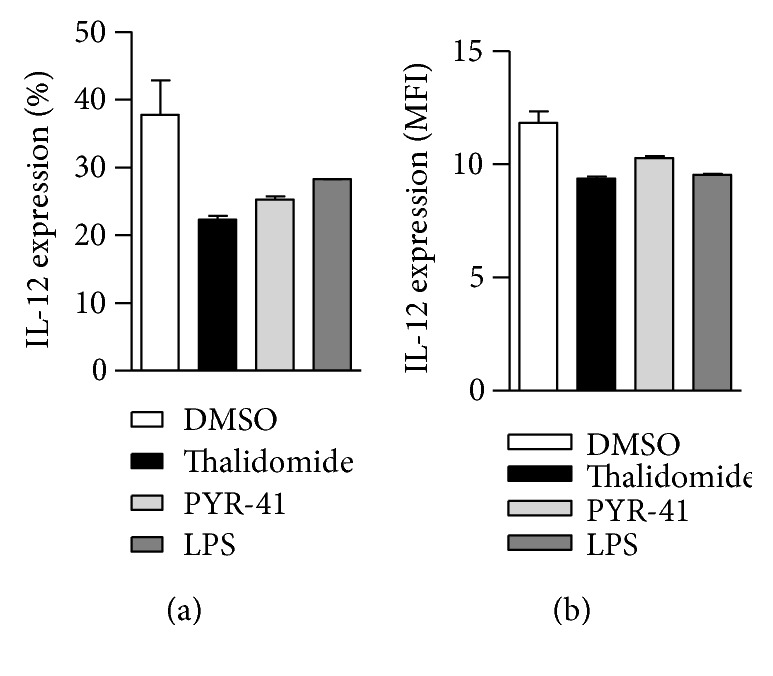
Treatment with PYR-41 or thalidomide has not increased IL-12 secretion. Murine bone marrow-derived DC (cultured for 4 d) conferred PYR-41 (5 *μ*M), thalidomide (30 *μ*M), LPS (10 ng/ml), or DMSO stimulation for 12 hrs, and IL-12 expression was determined by flow cytometry with intracellular IL-12 staining. The positive percentages (a) and the mean fluorescence intensity (b) of analyzed population were shown. Data were presented as the mean ± SEM and one-way ANOVA with Newman–Keuls post test. One representative from 3 independent experiments was shown.

To explore the effects of PYR-41 and thalidomide on cell viability, CCK-8 staining and PI staining were performed and cell viability was determined. Despite that PYR-41 and thalidomide impaired cell viability in a dose-dependent manner (Figure S1a), the dose used in these experiments had no effect on cell apoptosis (Figure S1b). Analyses of intracellular antigen in PYR-41- or thalidomide-treated DC revealed that these treatments had no effect on antigen uptake (Supplementary Materials description). As CD80 and CD86 are important costimulatory molecules in T cell-APC interaction [28], we further accessed the effects of PYR-41 and thalidomide on the expressions of CD80/CD86. Despite that long-term LPS stimulation increased CD80/CD86 expression (Figure 2), the treatment with PYR-41 or thalidomide had no effect on CD80/CD86 expression either in long-term- or in short-term-stimulated conditions (Figure 2), indicating that the treatment with PYR-41 or thalidomide has no effect on costimulatory signaling. Intracellular IL-12 staining also revealed that the treatment with PYR-41 or thalidomide did not increase IL-12 secretion (Figure 3), indicating that neither PRY-41 nor thalidomide has effect on cytokine signaling. All these observations demonstrate that the treatment with PYR-41 or thalidomide impairs DC cross-priming.

### 3.2. Treatment with PYR-41 or Thalidomide Inhibits DC Cross-Presentation

**Figure 4:**
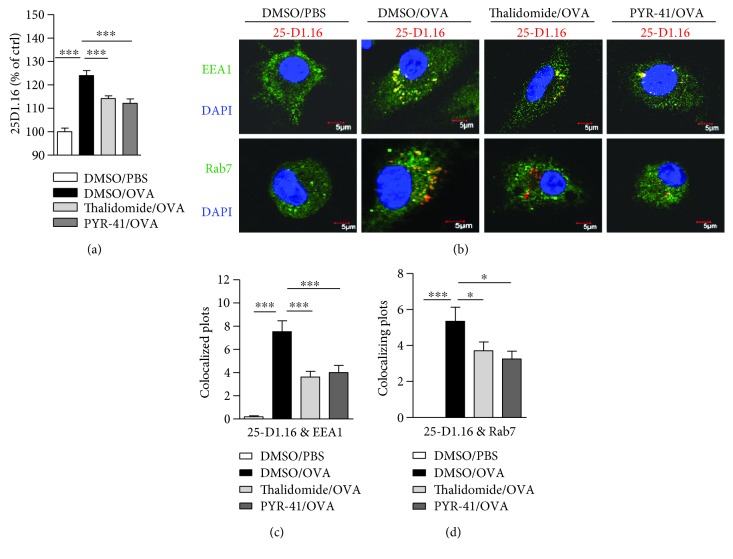
Treatment with PYR-41 or thalidomide inhibits DC cross-presentation. Murine bone marrow-derived DC (cultured for 4 d) conferred thalidomide (30 *μ*M), PYR-41 (5 *μ*M), or DMSO treatment prior to ovalbumin (50 *μ*g/ml) or PBS pulse. The effects of PYR-41 and thalidomide on cross-presentation were determined by flow cytometric analyses (a) and confocal microscope (b), respectively. For immunofluorescence observations, cross-presented OVA was stained with red (25-D1.16), EEA1 and Rab7 are all stained with green, and nuclei were counterstained with blue (DAPI). Original magnification, ×600. Data were presented as the mean ± SEM, ^∗^ *p* < 0.05, ^∗∗∗^ *p* < 0.001, and one-way ANOVA with Newman–Keuls post test. One representative from 3 independent experiments was shown.

As the treatment with PYR-41 or thalidomide impaired DC cross-priming (Figure 1) and had no effect on costimulator and cytokine signaling (Figures 2 and 3), we wonder whether the effects of PYR-41 and thalidomide on DC cross-priming were due to the impaired cross-presentation. Toward this end, DC were treated with PYR-41 and thalidomide and cross-presented ovalbumin was determined by flow cytometric analyzes with 25D1.16 antibody staining which is specific to SIINFEKL-H2Kb complex. The results were presented as mean of fluorescence intensity (MFI). The results showed that both PYR-41 and thalidomide inhibited DCs' ability of cross-presentation by revealing decreased SIINFEKL-H2Kb complex (Figure 4(a)). As endosome is being the compartment for DC cross-presentation [27], the effects of PYR-41 and thalidomide on cross-presented ovalbumin were reconfirmed by confocal microscope observation with endosomal marker EEA1/Rab7 and cross-presented ovalbumin antibody staining (Figures 4(b) and 4(d)). These observations demonstrated that treatment with PYR-41 and thalidomide impairs DC ability of cross-presentation.

### 3.3. Treatment with PYR-41 or Thalidomide Abolishes the Endosomal Recruitment of p97

**Figure 5:**
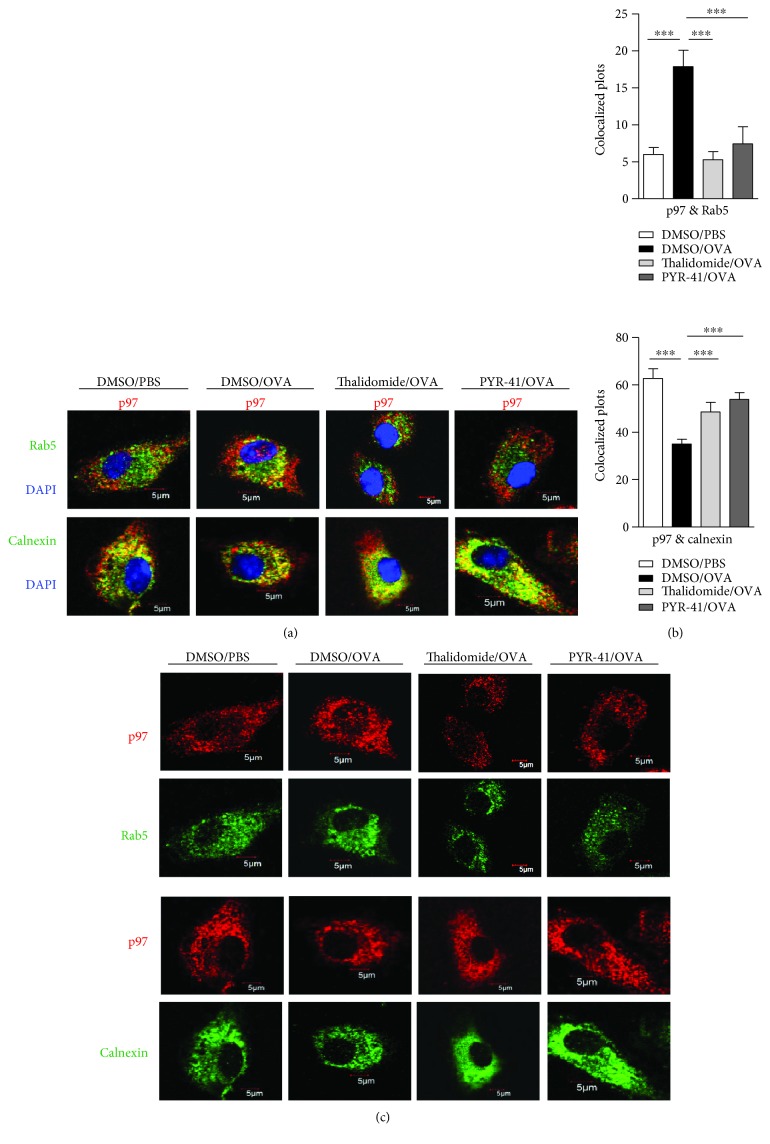
Treatment with PYR-41 or thalidomide abolishes the endosomal recruitment of p97. Murine bone marrow-derived DC (cultured for 4 d) firstly conferred thalidomide (30 *μ*M), PYR-41 (5 *μ*M), or DMSO treatment prior to ovalbumin (50 *μ*g/ml) or PBS pulse. The relocation of p97 from endoplasmic reticulum to endosomes (a) was determined by confocal microscope by Rab5, calnexin, and p97 antibody staining. The colocalized plots of p97 with Rab5 and calnexin (b) were counted and analyzed. The expressions of Rab5/p97 and calnexin/p97 in the cells which are corresponding to the (a) colocalized cells were shown (c). Nuclei were counterstained with DAPI (blue). Original magnification, ×600. Data were presented as the mean ± SEM, ^∗∗∗^ *p* < 0.001, and one-way ANOVA with Newman–Keuls post test. One representative from 3 independent experiments was shown. Rab5: early endosome marker; calnexin: endoplasmic reticulum marker.

In the process of cross-presentation, a group of proteins, such as p97, translocates to endosomes to facilitate the transport of internalized antigen from endosomes to cytosol [8, 13]. To verify the effects of PYR-41 and thalidomide on the endosomal relocation of p97, DC was treated with PYR-41 or thalidomide prior to ovalbumin pulse and the translocation of p97 from endoplasmic reticulum (ER) to endosomes was observed. Rab5 and calnexin stand for the marker of early endosomes and endoplasmic reticulum, respectively. As shown in Figure 5, compared to scramble, treatment with ovalbumin obviously increased the colocalized spots of p97 with Rab5 (Figures 5(a) and 5(b)), indicating that ovalbumin promotes p97 toward endosomes. Importantly, treatment with PYR-41 and thalidomide efficiently inhibited the colocalized spots of p97 with Rab5 (Figures 5(a) and 5(b)). As treatment with PYR-41, thalidomide, and ovalbumin did not affect the expressions of p97, Rab5, and calnexin (Figure 5(c)), the phenomena that PYR-41 and thalidomide decreased the translocation of p97 indicate that the inhibition of endosomal recruitment of p97 contributes to PYR-41- and thalidomide-impaired cross-presentation.

### 3.4. Treatment with PYR-41 or Thalidomide Abrogates the Endosomal Recruitment of Sec61

**Figure 6 fig6:**
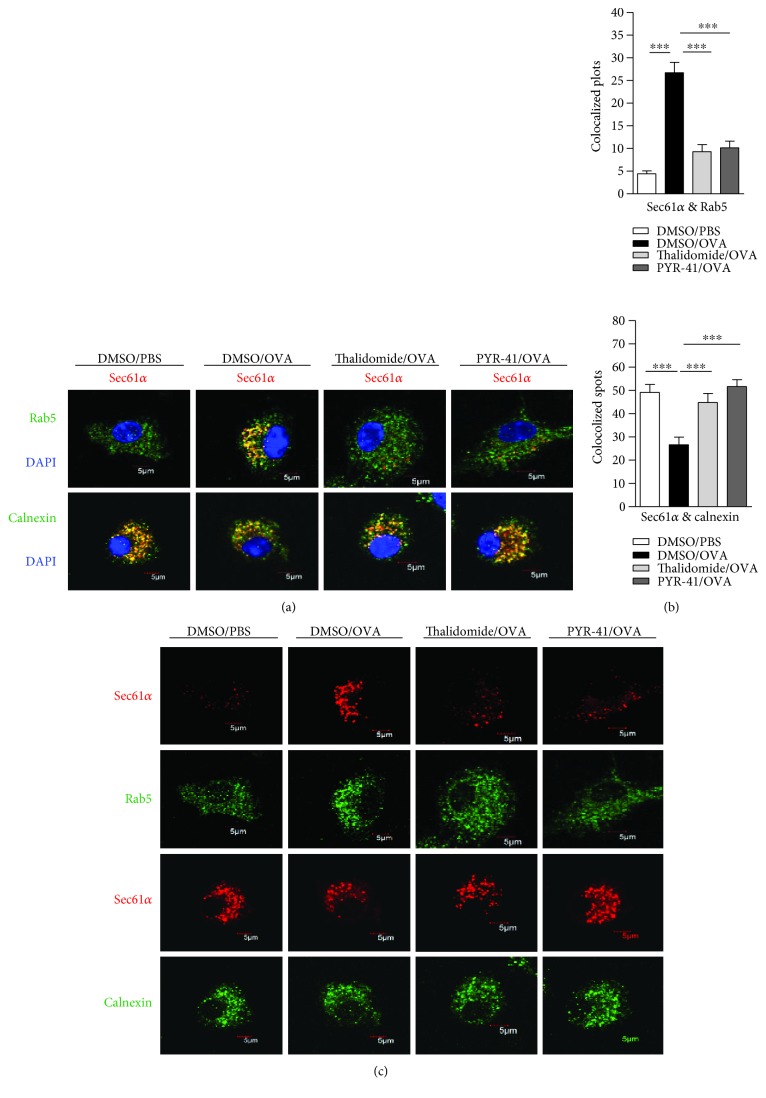
Treatment with PYR-41 or thalidomide abrogates the endosomal recruitment of Sec61*α*. Murine bone marrow-derived DC (cultured for 4 d) conferred thalidomide (30 *μ*M), PYR-41 (5 *μ*M), or DMSO treatment prior to ovalbumin (50 *μ*g/ml) or PBS pulse. The relocation of Sec61*α* from endoplasmic reticulum to endosomes was determined by confocal microscope by Rab5, calnexin, and Sec61*α* antibody staining. The colocalized plots of Sec61*α* (b) with Rab5/calnexin were counted and analyzed. The expressions of Rab5/Sec61*α* and calnexin/Sec61*α* in the cells which are corresponding to the (a) colocalized cells were shown (c). Nuclei were counterstained with DAPI (blue). Original magnification, ×600. Data were presented as the mean ± SEM, ^∗∗∗^ *p* < 0.001, and one-way ANOVA with Newman–Keuls post test. One representative from 3 independent experiments was shown. Rab5: early endosome marker; calnexin: endoplasmic reticulum marker.

**Figure 7:**
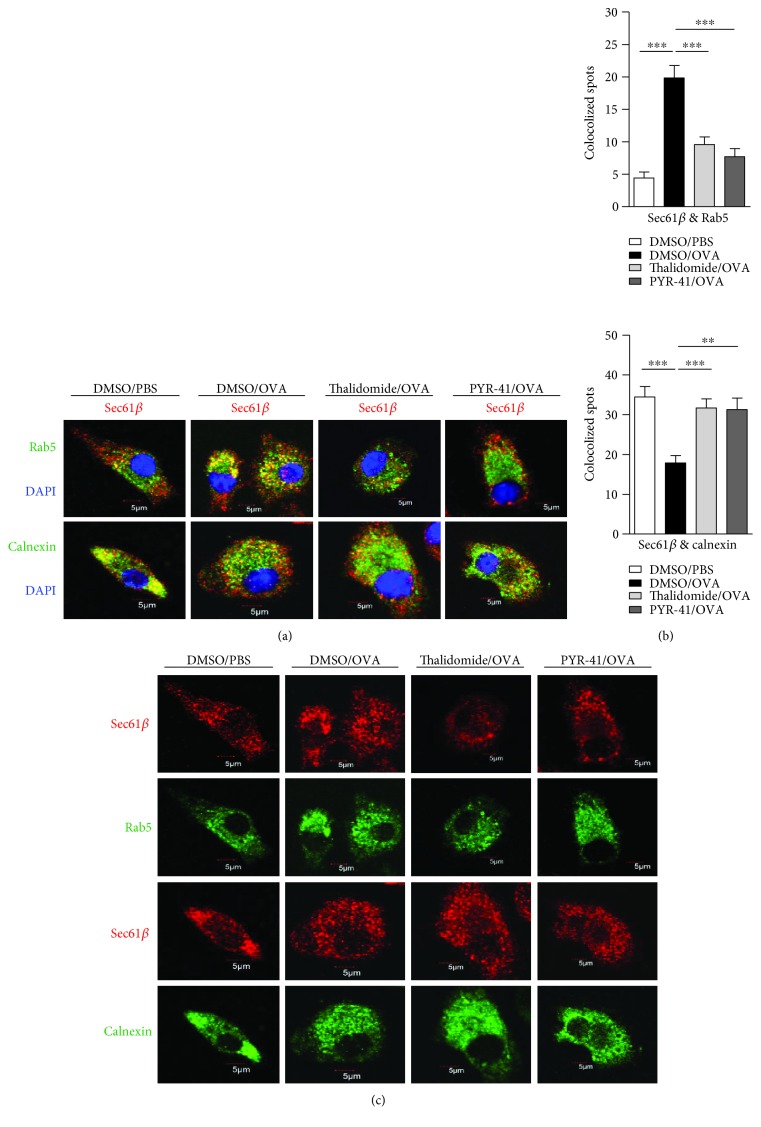
Treatment with PYR-41 or thalidomide abrogates the endosomal recruitment of Sec61*β*. Murine bone marrow-derived DC (cultured for 4 d) conferred thalidomide (30 *μ*M), PYR-41 (5 *μ*M), or DMSO treatment prior to ovalbumin (50 *μ*g/ml) or PBS pulse. The relocation of Sec61*β* (a) from endoplasmic reticulum to endosomes was determined by confocal microscope by Rab5, calnexin, and Sec61*β* antibody staining. The colocalized plots of Sec61*β* (b) with Rab5/calnexin were counted and analyzed. The expressions of Rab5/Sec61*β* and calnexin/Sec61*β* in the cells which are corresponding to the (a) colocalized cells were shown (c). Nuclei were counterstained with DAPI (blue). Original magnification, ×600. Data were presented as the mean ± SEM, ^∗∗^ *p* < 0.01, ^∗∗∗^ *p* < 0.001, and one-way ANOVA with Newman–Keuls post test. One representative from 3 independent experiments was shown. Rab5: early endosome marker; calnexin: endoplasmic reticulum marker.

It was documented that Sec61, another protein in the ER, translocates toward endosomes upon antigen pulse and provides energy for transmembrane movement of the antigen [14–16]. To explore the effects of PYR-41 and thalidomide on the endosomal recruitment of Sec61, the cells were treated with these drugs and the translocation of Sec61*α* and Sec61*β* from ER toward endosomes was observed. As shown in Figure 6, ovalbumin treatment increased the colocalized spots of Sec61*α* with Rab5 (Figures 6(a) and 6(b)) and inhibited the colocalized spots of Sec61*α* with calnexin (Figures 6(a) and 6(b)), indicating that ovalbumin promotes Sec61*α* translocation from ER to endosomes. Importantly, treatment with PYR-41 and thalidomide efficiently inhibited the colocalized spots of Sec61*α* with Rab5 and augmented the colocalized spots of Sec61*α* with calnexin (Figures 6(a) and 6(b)). The observation about the endosomal recruitment of Sec61*β* also derived a similar conclusion (Figure 7). As treatment with PYR-41, thalidomide, and ovalbumin did not affect the expressions of Sec61*α*, Sec61*β*, Rab5, and calnexin (Figures 6(c) and 7(c)), all these phenomena that PYR-41 and thalidomide decreased the translocation of Sec61*α* and Sec61*β* indicate that inhibition of the endosomal recruitment of Sec61*α* and Sec61*β* contributes to PYR-41- and thalidomide-impaired cross-presentation.

### 3.5. Treatment with PYR-41 or Thalidomide Inhibits LPS-Induced NF-*κ*B Activation

**Figure 8:**
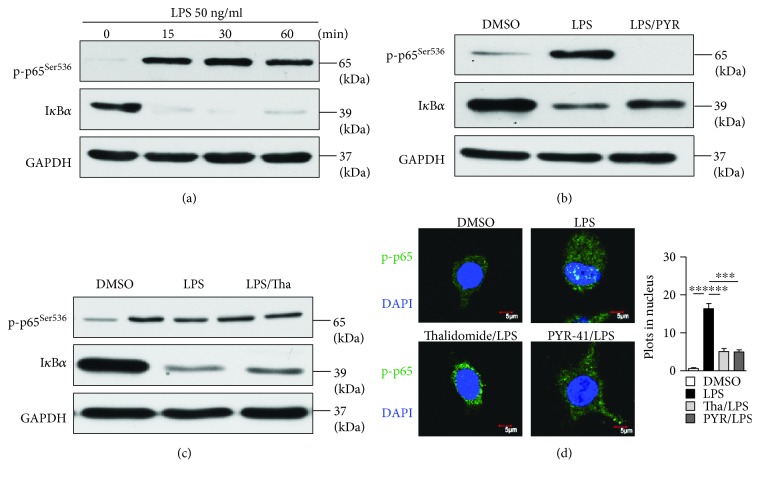
Treatment with PYR-41 or thalidomide inhibits LPS-induced NF-*κ*B activation. Murine bone marrow-derived DC (cultured for 4 d) conferred thalidomide (30 *μ*M), PYR-41 (5 *μ*M), or DMSO treatment prior to LPS (50 ng/ml) stimulation. (a–c) The effects of LPS, PYR-41, and thalidomide on NF-*κ*B activation were determined by Western blot by revealing I*κ*B*α* degradation and p65 phosphorylation. GAPDH was used as internal control. (d) The effects of LPS, PYR-41, and thalidomide on NF-*κ*B activation were determined by confocal microscope by revealing the nuclei relocation of phosphorylated p65. Nuclei were counterstained with DAPI (blue). Original magnification, ×600. Data were presented as the mean ± SEM, ^∗∗∗^ *p* < 0.001, and one-way ANOVA with Newman–Keuls post test. One representative from 3 independent experiments was shown.

Our previous study showed that TLR4-MyD88-IRAK4-dependent TAP translocation from endoplasmic reticulum toward endosomes facilitates DC cross-presentation [18]. Other reports also demonstrated that the endosomal recruitment of Sec61, which depends on NF-*κ*B activation, is responsible for cross-presentation [15]. Hence, we wonder whether PYR-41- impaired cross-presentation and thalidomide-impaired cross-presentation were due to the inhibition of TLR4-induced NF-*κ*B activation. Toward this end, the cells were treated with PYR-41 or thalidomide prior to LPS stimulation and the activation of NF-*κ*B was determined. As the evidence of NF-*κ*B activation, LPS treatment efficiently induced the phosphorylation of p65 and I*κ*B*α* degradation (Figure 8(a)). The pretreatment with PYR-41 or thalidomide not only inhibited p65 phosphorylation but also attenuated I*κ*B*α* degradation (Figures 8(b) and 8(c)). The confocal microscope also showed that nucleus translocation of phosphorylated p65 was abolished by the treatment with PYR-41 or thalidomide (Figure 8(d)). All these observations indicate that PYR-41 and thalidomide inhibit LPS-induced NF-*κ*B signaling.

### 3.6. Treatment with PYR-41 or Thalidomide Inhibits LPS-Induced Myddosome Formation

**Figure 9:**
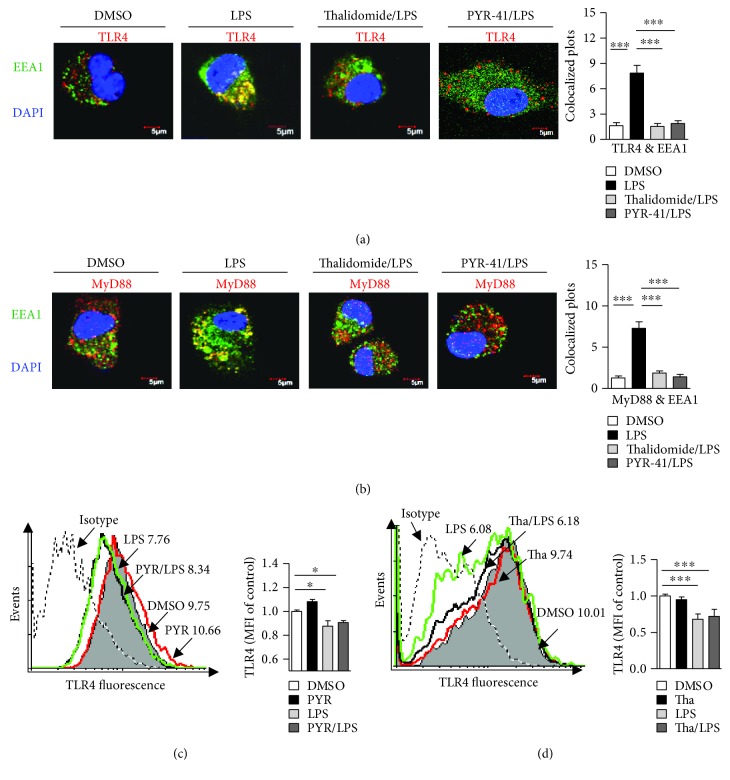
Treatment with PYR-41 or thalidomide inhibits LPS-induced myddosome formation. Murine bone marrow-derived DC (cultured for 4 d) conferred thalidomide (30 *μ*M), PYR-41 (5 *μ*M), or DMSO treatment prior to LPS (50 ng/ml) stimulation. The relocation of TLR4 (a) and MyD88 (b) was determined by confocal microscope by EEA1, TLR4, and MyD88 antibody staining. Nuclei were counterstained with DAPI (blue). Original magnification, ×600. The colocalized plots of TLR4 (a) or MyD88 (b) with EEA1 were counted and analyzed. (c–d) The effects of thalidomide and PYR-41 on TLR4 expression on DC were determined via flow cytometry. Numbers in histogram indicate MFI of analyzed population. The data were presented as the mean ± SEM, ^∗^ *p* < 0.05, ^∗∗∗^ *p* < 0.001, and one-way ANOVA with Newman–Keuls post test.

TLR4, as an essential signal pathway of pattern recognition receptor, could be internalized by LPS-stimulated cells and promote myddosome formation, which facilitate the endosomal recruitments of TAP and Sec61 [15, 18, 20, 21]. As PYR-41 and thalidomide inhibited the endosomal recruitments of p97/Sec61 (Figures 5 [Fig fig6]–7) and attenuated NF-*κ*B activation (Figure 8), we wonder whether PYR-41- impaired cross-presentation and thalidomide-impaired cross-presentation were due to the inhibition of myddosome formation. Toward this end, the cells were pretreated with PYR-41 or thalidomide and endosomal recruitment of TLR4 and MyD88 was observed. As the evidence of myddosome formation, LPS stimulation obviously augmented the colocalized spots of TLR4 and MyD88 with EEA1 (Figures 9(a) and 9(b)). The pretreatment with PYR-41 or thalidomide not only inhibited the colocalized spots of TLR4 with EEA1 (Figure 9(a)) but also attenuated the colocalized spots of MyD88 with EEA1 (Figure 9(b)). While LPS stimulation decreased the existence of TLR4 in the DC surface, treatment with thalidomide and PYR-41 had no effect on TLR4 expression on the DC surface (Figures 9(c) and 9(d)). As EEA1 is the marker of early endosome, the above observations indicate that the inhibition of myddosome formation contributes to PYR-41- and thalidomide-impaired cross-presentation.

## 4. Discussion

**Figure 10:**
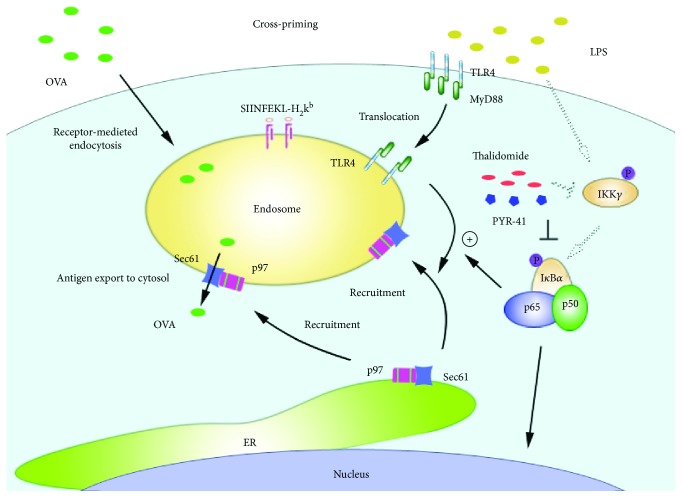
Mechanism of PYR-41- and thalidomide-impaired DC cross-presentation. PYR-41 and thalidomide inhibit myddosome formation via reducing NF-*κ*B activation. Decreased myddosome formation recruited less p97 and Sec61 from the ER toward the endosomes, leading to less internalized antigens exported to the cytosol to be degraded, resulting in a reduced cross-presentation and subsequent cross-priming.

In this study, we investigated the effects of PYR-41 and thalidomide on DC cross-presentation. We demonstrated that treatment with PYR-41 or thalidomide attenuates NF-*κ*B activation. Concurrently, the decreased formation of myddosome, together with inhibited endosomal recruitment of p97 and Sec61, leads to PYR-41- and thalidomide-induced the impairment of cross-presentation and cross-priming (Figure10).

Cross-presentation refers to MHC I molecules binding to extracellular antigen [7]. This process is of particular importance, because it permits the presentation of exogenous antigens, which are normally presented by MHC II on the surface of infected DC [9]. In our previous studies, ovalbumin was used to pulse DC and revealed that *α*7 nAChR activation increases DC cross-presentation [18, 24]. SIINFEKL peptide, which corresponds to ovalbumin 257-264 and directly binds to MHC class I molecules, is an intracellular model antigen for the MHC I presentation pathway [29]. Hence, the whole extracellular ovalbumin protein, instead of SIINFEKL peptide, was used to pulse DC and SIINFEKL-H2Kb complex was determined to assess the efficiency of cross-presentation [8, 15, 19].

Cross-presentation mainly occurs in the endosome-to-cytosol pathway, which needs TLR4-MyD88 signal to mediate the endosomal relocation of ER-derived components [17–19]. The endosomal recruitment of p97, which depends on mannose receptor, was demonstrated to facilitate DC cross-presentation [8]. On the other hand, NF-*κ*B activation-dependent endosomal relocation of Sec6 was also documented to be needed for DC cross-presentation [15]. Our previous study further revealed that the endosomal TAP relocation depends on TLR4-MyD88-IRAK4 signaling [18]. Hence, despite that NF-*κ*B inactivation-decreased myddosome formation and the inhibition of endosomal recruitment of p97/Sec61 were verified to contribute to PYR-41- and thalidomide-impaired cross-presentation, the exact effects of PYR-41 and thalidomide on the endosomal recruitment of TAP are still uncertain and need further explorations.

In the endosome-to-cytosol pathway, antigen degradation occurring in the proteasomes indicates that antigens internalized in endosomes need to be transported from the compartment to the cytosol [12]. Sec61 was reported to be recruited by TLR4 signaling from the ER to the endosomes and facilitated the transport of internalized antigen [15]. It was not unpredicted to find that the endosomal relocation of Sec61 was indeed inhibited by the treatment with PYR-41 and thalidomide (Figures 6 and 7). The endosomal recruitment of p97 was also found to facilitate the cytosolic access of several different antigens [30]. As the translocation of p97 toward endosomes was mediated by the ubiquitination of mannose receptor [8], the exact effects and the underlying mechanism of PYR-41 and thalidomide on ubiquitination of mannose receptor need further elucidation.

Ubiquitination, which is an important event for protein relocation and degradation, is emerging as a new mechanism for immune regulation [31]. For example, the ubiquitination of mannose receptor mediates the endosomal relocation of p97 and promotes the cytosolic translocation of antigens [8]. On the other hand, TLR4-MyD88-IRAK4 signaling-induced NF-*κ*B activation was demonstrated to be necessary for the endosomal relocation of TAP [18, 19]. In the present study, despite that NF-*κ*B inactivation and the decreased endosomal translocation of TLR4 and MyD88 were achieved by treatment with thalidomide and PYR-41, the exact mechanism of myddosome mediating the endosomal recruitment of p97 and Sec61 still needs further investigation.

As antigen-presenting cells, in addition to cross-presentation, DC can also present antigen to T cells by classical MHC I-restricted endogenous antigen presentation and MHC II-restricted exogenous antigen presentation. Using DO11.1 cells that respond to presentation of OVA presented by I-A^d^, Deng et al. found that thalidomide inhibits tumor necrosis factor-alpha production and I-A^d^ restricted antigen presentation [32]. Chen et al found that PYR41 inhibits DC-induced T cell proliferation with decreased expression of CD69 and CD25 [33]. In the present study, despite that we found thalidomide- and PYR-41-inhibited DC cross-presentation, the exact effects of thalidomide and PYR-41, especially PYR-41, on MHC II-restricted antigen presentation are still unknown and need further investigation.

Documented data showed that the purity, phenotypes, culturing condition, and cell validation are critical for dendritic cell study [23, 25–27, 34]. In the present study, the DC was derived from the bone marrow with GM-CSF (30 ng/ml) and IL-4 (1 ng/ml) and cultured for 4 days [22]. In that condition without any LPS contamination, the DC was relatively immature DC or semimature DC [22, 23, 34]. Hence, despite that the effects of PYR-41 and thalidomide on semimature DC were verified, the effects of these drugs on DC maturation, phenotypes, and culturing condition need further investigation.

## 5. Conclusions

Our data provide a new insight for PYR-41 and thalidomide in cross-presentation and cross-priming, which is mediated by the combined action of decreased MyD88 translocation and the inhibition of endosomal recruitments of p97 and Sec61 (Figure 10). These observations suggest that NF-*κ*B signaling and p97 and Sec61 molecules might be potential candidates for dealing with the therapeutic side effects of PYR-41 and thalidomide.

## Supplementary Materials

Supplementary Materials The effects of PYR-41 and thalidomide on cell viability. Murine bone marrow-derived DC (cultured for 4 d) conferred thalidomide, PYR-41, cisplatin (4 *μ*g/ml), or DMSO treatment, and cell viability was determined by CCK-8 assays. The results showed that while cisplatin efficiently decreased DC's viability, thalidomide treatment with 5, 15 and 30 *μ*M concentration had no effect on DC's viability. Contract to thalidomide, the results of PYR-41 treatment revealed that 15 and 30 *μ*M, but not 5 *μ*M, obviously decreased DC's viability. Raw264.7 cells conferred treatment with PYR-41 (5 *μ*M), thalidomide (30 *μ*M), cisplatin (4 *μ*g/ml), or DMSO for 12 hrs, and the cell viability was determined by flow cytometric analyses with propidium iodide (PI) staining. The results revealed that cisplatin, but not PYR-41 and thalidomide, obviously induced cell apoptosis. The concentration of PYR-41 and thalidomide used in this study had no effect on DC endocytosis. Murine bone marrow-derived DC (cultured for 4 d) conferred PYR-41 (5 *μ*M), thalidomide (30 *μ*M), or DMSO treatment prior to 15 min ovalbumin FITC (5 *μ*g/ml) pulse at 37°C. The cells incubated with ovalbumin FITC at 4°C were used as negative control. The effect of PYR-41 and thalidomide on cell endocytosis was determined by flow cytometry. The results showed that, while DMSO treatment reveals 6.83% ovalbumin-positive cells, treatment with PYR-41 and thalidomide achieved 5.89% and 5.82% ovalbumin-positive cells, indicating that the concentration of PYR-41 and thalidomide used in this study had no effect on DC endocytosis.
