# Supplementary material for: PYR-41 and Thalidomide Impair Dendritic Cell Cross-Presentation by Inhibiting Myddosome Formation and Attenuating the Endosomal Recruitments of p97 and Sec61 via NF-κB Inactivation
Source: J Immunol Res. 2018 Jul 5;2018:5070573. doi: 10.1155/2018/5070573 (PMC6057288; doi:10.1155/2018/5070573)
Supplement: Supplementary Materials — The effects of PYR-41 and thalidomide on cell viability. Murine bone marrow-derived DC (cultured for 4 d) conferred thalidomide, PYR-41, cisplatin (4 μg/ml), or DMSO treatment, and cell viability was determined by CCK-8 assays. The results showed that while cisplatin efficiently decreased DC's viability, thalidomide treatment with 5, 15 and 30 μM concentration had no effect on DC's viability. Contract to thalidomide, the results of PYR-41 treatment revealed that 15 and 30 μM, but not 5 μM, obviously decreased DC's viability. Raw264.7 cells conferred treatment with PYR-41 (5 μM), thalidomide (30 μM), cisplatin (4 μg/ml), or DMSO for 12 hrs, and the cell viability was determined by flow cytometric analyses with propidium iodide (PI) staining. The results revealed that cisplatin, but not PYR-41 and thalidomide, obviously induced cell apoptosis. The concentration of PYR-41 and thalidomide used in this study had no effect on DC endocytosis. Murine bone marrow-derived DC (cultured for 4 d) conferred PYR-41 (5 μM), thalidomide (30 μM), or DMSO treatment prior to 15 min ovalbumin FITC (5 μg/ml) pulse at 37°C. The cells incubated with ovalbumin FITC at 4°C were used as negative control. The effect of PYR-41 and thalidomide on cell endocytosis was determined by flow cytometry. The results showed that, while DMSO treatment reveals 6.83% ovalbumin-positive cells, treatment with PYR-41 and thalidomide achieved 5.89% and 5.82% ovalbumin-positive cells, indicating that the concentration of PYR-41 and thalidomide used in this study had no effect on DC endocytosis. [file 5070573.f1.doc]

**
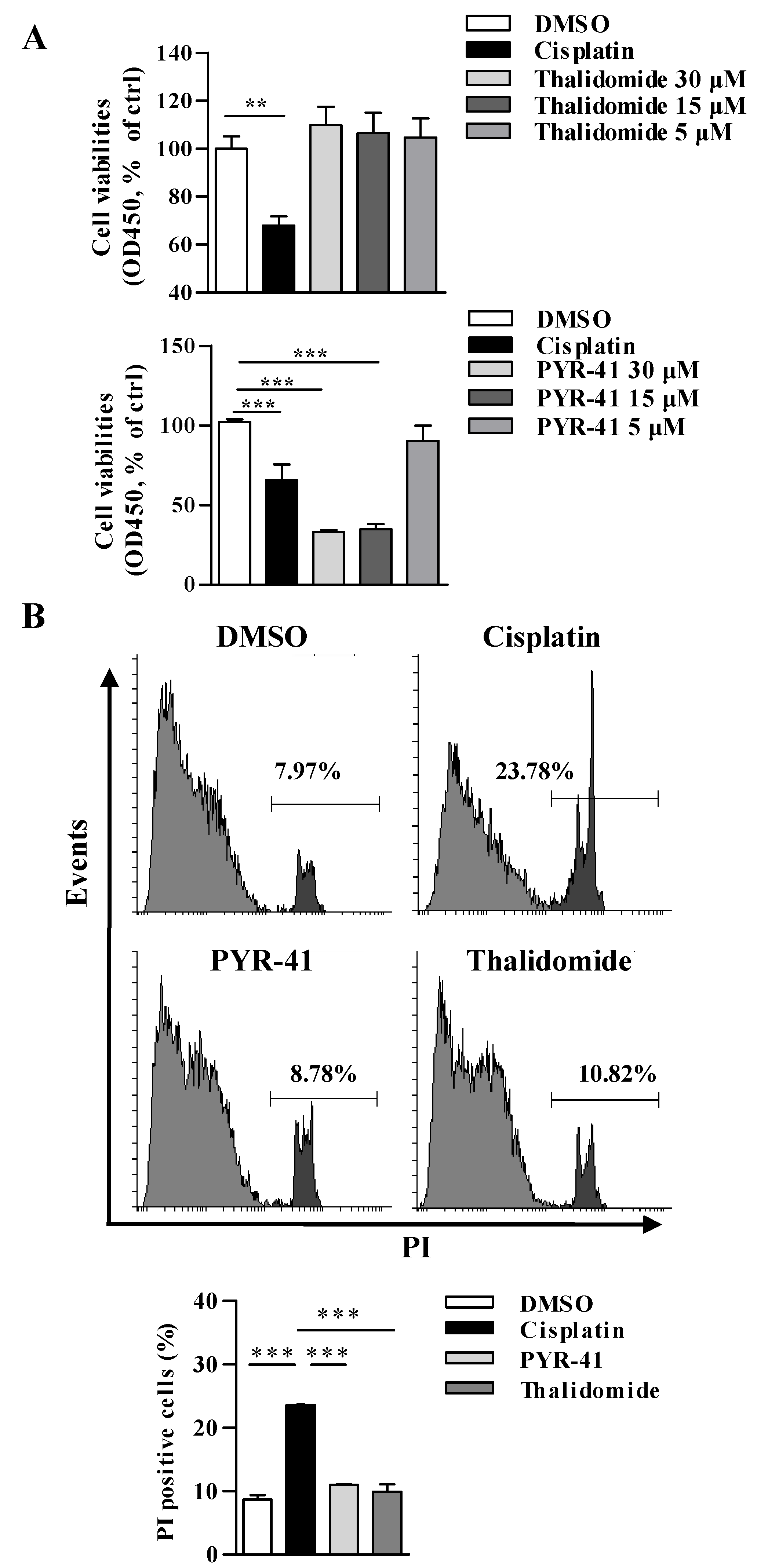
**

**S.Figure 1: The effects of PYR-41 and thalidomide on cell viability.** (A). Murine bone marrow-derived DC (cultured for 4 d) conferred thalidomide, PYR-41, cisplatin (4 μg/ml) or DMSO treatment and cell viabiliy was determined by CCK-8 assays. (B). Raw264.7 cells were treated with PYR-41 (5 μM), thalidomide (30 μM), cisplatin (4 μg/ml) or DMSO for 12 hrs and cell apoptosis was determined by flow cytometry with propidine iodide (PI) staining. Numbers in histogram indicate the positive percentage of analyzed population. Cisplatin was used as positive control. Data were presented as mean ± SEM, ** p<0.01, *** p<0.001, one-way ANOVA with Newman-Keuls post test. One representative from 3 independent experiments was shown.

**
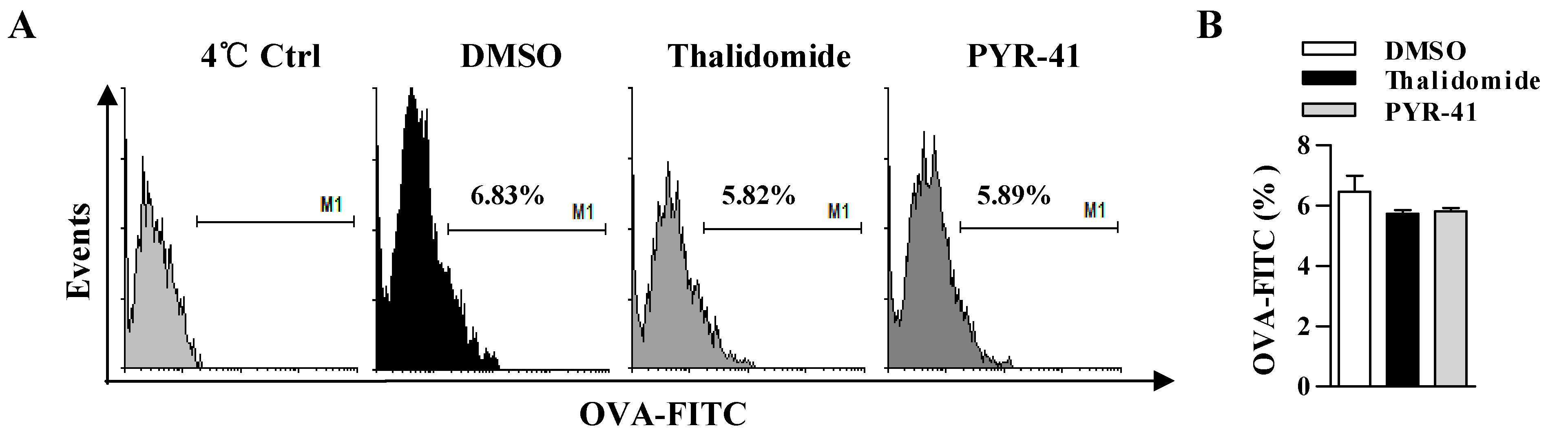
**

**S.Figure 2: The effects of PYR-41 and thalidomide on DC endocytosis.** (A). Murine bone marrow-derived DC (cultured for 4 d) conferred PYR-41 (5 μM), thalidomide (30 μM) or DMSO treatment prior to 15 min ovalbumin-FITC (5 μg/ml) pulse at 37℃. The cells incubated with ovalbumin-FITC at 4℃ were used as negative control. Cell endocytosis was determined by flow cytometry. (B). Statistic figure of flow cytometry. Numbers in histogram indicate the positive percentage of analyzed population. Data were presented as mean ± SEM, one-way ANOVA with Newman-Keuls post test. One representative from 3 independent experiments was shown.
